# Supplementary material for: Pediatric Inflammatory Bowel Disease Tissue Classification From Pathology Slide Images: Detecting Phenotypes Using Computer Vision
Source: Gastro Hep Adv. 2026 Feb 14;5(5):100899. doi: 10.1016/j.gastha.2026.100899 (PMC13022611; doi:10.1016/j.gastha.2026.100899)
Supplement: Supplementary Table 1 [file mmc1.pdf]

Supplemental table 1 Interobserver rater results and interobserver reliability for sections in additionally labelled subset

		**Rater 2**	
**Abnormal vs normal**		Abnormal	Normal
**Rater 1**	Abnormal	158	1
	Normal	4	11
**Active inflammation**		Present	Absent
**Rater 1**	Present	142	1
	Absent	14	17
**Chronic changes**		Present	Absent
**Rater 1**	Present	105	12
	Absent	15	42

	***κ* (95% confidence interval)**
**Abnormal vs. normal**	0.799 (0.626, 0.973)
**Active inflammation**	0.648 (0.477, 0.818)
**Chronic changes**	0.643 (0.519, 0.767)

κ: Cohen's Kappa Statistic
