# Supplementary material for: Pediatric Inflammatory Bowel Disease Tissue Classification From Pathology Slide Images: Detecting Phenotypes Using Computer Vision
Source: Gastro Hep Adv. 2026 Feb 14;5(5):100899. doi: 10.1016/j.gastha.2026.100899 (PMC13022611; doi:10.1016/j.gastha.2026.100899)
Supplement: Supplementary Table 2 [file mmc2.pdf]

Supplemental Table 2 Number of tissue section scans in each cohort

	**Fold 1**		**Fold 2**		**Fold 3**		**Fold 4**		**Fold 5**	
	**Train/Val**	**Test**	**Train/Val**	**Test**	**Train/Val**	**Test**	**Train/Val**	**Test**	**Train/Val**	**Test**
**Total scans**	1018 (0.78)	284 (0.22)	1062 (0.82)	240 (0.18)	1093 (0.84)	209 (0.16)	1002 (0.77)	300 (0.23)	1033 (0.79)	269 (0.21)
**Normal**	614 (0.60)	152 (0.54)	602 (0.57)	164 (0.68)	651 (0.60)	115 (0.55)	591 (0.59)	175 (0.58)	606 (0.59)	160 (0.59)
**Abnormal**	404 (0.40)	132 (0.46)	460 (0.43)	76 (0.32)	442 (0.40)	94 (0.45)	411 (0.41)	125 (0.42)	427 (0.41)	109 (0.41)
**No inflam**	657 (0.65)	177 (0.62)	669 (0.63)	165 (0.69)	713 (0.65)	121 (0.58)	641 (0.64)	193 (0.64)	656 (0.64)	178 (0.66)
**Inflam**	361 (0.35)	107 (0.38)	393 (0.37)	75 (0.31)	380 (0.35)	88 (0.42)	361 (0.36)	107 (0.36)	377 (0.36)	91 (0.34)
**No changes**	685 (0.67)	191 (0.67)	705 (0.66)	171 (0.71)	754 (0.69)	122 (0.58)	675 (0.67)	201 (0.67)	685 (0.66)	191 (0.71)
**Changes**	333 (0.33)	93 (0.33)	357 (0.34)	69 (0.29)	339 (0.31)	87 (0.42)	327 (0.33)	99 (0.33)	348 (0.34)	78 (0.29)
