# Supplementary material for: Pediatric Inflammatory Bowel Disease Tissue Classification From Pathology Slide Images: Detecting Phenotypes Using Computer Vision
Source: Gastro Hep Adv. 2026 Feb 14;5(5):100899. doi: 10.1016/j.gastha.2026.100899 (PMC13022611; doi:10.1016/j.gastha.2026.100899)
Supplement: Extended PDF [file mmc3.pdf]

Gastro Hep Advances 2026;5:100899

ORIGINAL RESEARCH—CLINICAL

Pediatric Inflammatory Bowel Disease Tissue Classification From Pathology Slide Images: Detecting Phenotypes Using Computer Vision

Chloe Martin-King, PhD,[1] Ali Nael,[2,3] Louis Ehwerhemuepha,[1,4,5] Blake Calvo,[1,4] Quinn Gates,[1,4] Jamie Janchoi,[1] Elisa Ornelas,[1] Melissa Perez,[6] Andrea Venderby,[1,4] John Miklavcic,[4,7] Peter Chang,[8,9,10] Aaron Sassoon,[2] Brian Rubio,[11] Ghislaine Barragan,[12] and Kenneth Grant[6,13]

[1]Research Institute, Children's Health Orange County (CHOC), Orange, California; [2]Department of Pathology, CHOC, Orange, California; [3]Department of Pathology, University of California-Irvine (UCI) Medical Center, Orange, California; [4]Schmid College of Science and Technology, Chapman University, Orange, California; [5]Department of Statistics, UCI Donald Bren School of Information and Computer Sciences, Irvine, California; [6]Department of Gastroenterology and Nutrition, CHOC, Orange, California; [7]School of Pharmacy, Chapman University, Irvine, California; [8]Center for Artificial Intelligence in Diagnostic Medicine (CAIDM), UCI, Irvine, California; [9]Department of Radiological Sciences, UCI School of Medicine, Orange, California; [10]Department of Computer Science, UCI Donald Bren School of Information and Computer Sciences, Irvine, California; [11]College of Natural Sciences and Mathematics, California State University, Fullerton, California; [12]Department of Neuroscience, Cognition and Behavior, College of Arts and Sciences, University of San Diego, San Diego, California; and [13]Department of Pediatrics, UCI School of Medicine, Orange, California

BACKGROUND AND AIMS: With the advent of computer vision algorithms, we hypothesize that histopathology images from endoscopic biopsies may be utilized for automated classification of histologic phenotypes, thus guiding Crohn's disease and ulcerative colitis diagnosis and treatment. The aim of our study is to assess whether artificial intelligence can be used to improve pediatric inflammatory bowel disease outcomes by aiding pathologists with accurate detection of abnormal tissue sections. **METHODS:** Three two-dimensional (2D) convolutional neural networks with multiple instance learning were developed to classify histopathology tissue sections as normal vs abnormal and as containing active inflammation and/or chronic changes/architectural distortion. **RESULTS:** The abnormal vs normal classification model achieved an accuracy of 0.84, an area under the receiver operating characteristic curve (AUC-ROC) of 0.91, and an F1-score of 0.79. Precision, sensitivity, and specificity were 0.85, 0.74, and 0.91, respectively. The accuracy for predicting active inflammation was 0.85, AUC-ROC was 0.92, and F1-score was 0.78. The accuracy for predicting chronic changes/architectural distortion was 0.86, with an AUC-ROC of 0.93 and an F1-score of 0.76. All 3 models achieved a Matthews correlation coefficient of 0.67. **CONCLUSION:** The findings resulting from this study are significant primarily because they indicate that there is a strong artificial intelligence–interpretable signal present in endoscopic whole slide imaging, even with the necessary, weakly supervised method of multiple instance learning.

Keywords: Artificial Intelligence; Computer Vision; Histopathology; Multiple Instance Learning; Pediatric Inflammatory Bowel Disease; Whole Slide Imaging

Introduction

Many factors, including genetic, demographic, and environmental variables, may contribute to the phenotype of an inflammatory bowel disease (IBD) patient.[1–6] Differentiating Crohn's disease (CD) and ulcerative colitis (UC) can be difficult and is meaningful in the setting of multiple new treatment modalities, which may lead to appropriate intervention and improved quality of life. The increasing number of patients presenting at a very young age poses additional unique challenges to diagnosis and treatment.[7,8]

Histopathology from endoscopic biopsies is a standard component of IBD diagnostic evaluation. Findings may include architectural and inflammatory changes, the location and degree of discontinuity in different intestinal segments, and the presence of isolated epithelioid well-formed noncaseating granuloma unrelated to crypt distortion.[9–11] Certain features may be characteristic of either CD or UC. Proximal bowel mucosal histologic abnormalities point to a diagnosis of CD. Ileocolonic abnormalities are

Abbreviations used in this paper: **AI, artificial intelligence; AUC-ROC, area under the receiver operating characteristic curve; CD, Crohn's disease; CNN, convolutional neural network; IBD, inflammatory bowel disease; MCC, Matthews correlation coefficient; MIL, multiple instance learning; ROI, region of interest; UC, ulcerative colitis; WSI, whole slide image.**

Most current article

Copyright © 2026 The Authors. Published by Elsevier Inc. on behalf of the AGA Institute. This is an open access article under the CC BY license (http://creativecommons.org/licenses/by/4.0/).
2772-5723
https://doi.org/10.1016/j.gastha.2026.100899

also common in CD. Colorectal mucosal histologic abnormalities including heavy and widespread inflammatory cell infiltration, distorted and atrophic crypts, or surface erosions are indicative of UC.[9–11]

With the advent of computer vision algorithms, we hypothesize that histopathology images from endoscopic biopsies may be utilized for automated classification of histologic phenotypes. Visual signals within the slide images may be exploited by artificial intelligence (AI) algorithms to detect histologic features that guide CD and UC diagnosis. Histologic features are important when determining diagnosis and treatment, and many IBD studies that utilize whole slide images (WSIs) present promising results.[12–15]

The aim of our study is to assess whether AI can be used to improve pediatric IBD patient outcomes by providing pathologists with accurate detection of abnormal tissue sections in WSIs from endoscopic biopsies for classifying UC vs CD. A set of AI models was developed for detecting abnormal tissue, active inflammation, and chronic changes/architectural distortion in histopathology slide imaging.

Table 1. Patient Demographics

Variable	n (%) or median (IQR)
Sex	
Female	11 (44%)
Male	14 (56%)
Ethnicity	
Hispanic or Latino	6 (24%)
Not Hispanic or Latino	19 (76%)
Diagnosis	
Crohn's disease	10 (40%)
Ulcerative colitis	15 (60%)
Age in y at biopsy	12.9 (10, 15.1)

IQR, interquartile range.

Materials and Methods

Patient Selection

This retrospective study is considered minimal risk and was approved by Children's Health Orange County's in-house institutional review board, IRB number 2111186. A waiver of informed consent was granted. Patients diagnosed with IBD, less than 22 years of age, and who underwent endoscopic biopsy during the period from 2014 to 2022, were considered for this study. A random sample of 25 patients was included in this study due to the complexity of digitally capturing endoscopy biopsies. Patient demographics are listed in Table 1.

Histopathology Slide Scanning

All tissue sections from all slides associated with each patient's endoscopy visit were scanned. Samples were taken from along the entire digestive tract and included the following: duodenum, stomach, esophagus, ileum, cecum, colon, and rectum. Slides were scanned using a MikroScan digital pathology scanner at 40$\times$ magnification, 0.227–0.258 μm per pixel resolution. All tissue sections on each slide were scanned individually with the associated computer user interface by creating a bounding rectangle around each individual tissue section. At least 3 focal points were added to each rectangle prior to scanning to focus the microscope on the tissue and avoid interference from artifacts. The resulting dataset consists of 1302 tissue section scans taken from 229 whole slides.

Slide and Tissue Classification

Each slide was associated with a biopsy site and was labeled as normal or abnormal per the surgical pathology report. Therefore, slide classification was determined by the pathologist assigned to that patient's case. Each tissue section scan from an abnormal slide was correspondingly labeled as normal or abnormal by an expert pathologist. Abnormal sections were further labeled by the same pathologist as containing one or more of the following phenotypes: active inflammation, chronic changes/architectural distortion, and/or granulomas. These phenotypes are not mutually exclusive within the tissue; all may exist within the same tissue slice. Definitions for all class labels can be found in Martin-King et al.[16] For the remainder of this manuscript, the term "chronic changes" is used to indicate chronic changes/architectural distortion.

The presence of granulomas in histopathological analysis is an important indicator of CD. Granuloma is present in approximately 40% of children with CD.[9] We found that of the 10 patients with CD in our dataset, 5 had tissue sections that contained granuloma. However, there were only 42 (7.8% of the abnormal sections and 3.2% of the total number of sections) that contained granuloma. This extreme class imbalance made it infeasible to create a predictive model for granuloma classification using this dataset. Only active inflammation and chronic changes are addressed in the remainder of this manuscript. Of the 536 abnormal sections, 23 contained neither active inflammation nor chronic changes.

Interobserver Reliability for Section Classification

An additional pathologist labeled a subset of 9 patients for comparison. A total of 174 sections from 87 abnormal slides were labeled by the second pathologist. Cohen's kappa,[17] κ, was utilized to measure the interobserver reliability score for this subset. Possible κ values range from -1 to 1, with values $>$0.4–0.6 indicating moderate agreement, $>$0.6–0.8 indicating substantial agreement, and $>$0.8–1 indicating almost perfect or perfect agreement.[18] Interobserver rater results and interobserver reliability scores with 95% confidence intervals for each phenotype are provided in Supplementary Table 1.

Preprocessing

Due to the large size of each tissue section scan (median size of 28,416 by 28,928 pixels with 3 color channels), the scans were subdivided into 4096 $\times$ 4096 patches with 25% overlap along the x and y axes. Patches were resized to 128 $\times$ 128 pixels using bilinear interpolation to reduce computational cost during model development and save memory resources. Zero-padding was applied to scans with dimensions not evenly divisible by 4096 to ensure that the entire scan could be considered for inclusion in the dataset. Thresholding

techniques, including Otsu's method, struggled to indicate uninformative patches when they contained pervasive air-bubble or pen-mark artifacts. Therefore, an intermediate convolutional neural network (CNN), which was trained on a subset of patches, was utilized to remove patches containing pervasive artifacts or slide background. Otsu's thresholding method was implemented following the CNN to remove patches with insufficient tissue (less than 20% tissue) from the dataset. A random sample of the remaining image patches was surveyed to visually check for misidentified image patches. Due to the small number of patients, we opted to diminish noise by manually removing patches that were incorrectly selected for model building within this subset. 24,372 patches were included in model training/validation and testing.

Table 2 lists the counts for slides, sections, and patches, as well as parses by class and subclass. Figure 1 shows a visual distinction between whole slide, tissue sections, and patches.[16]

We chose to forgo applying normalization to images before training due to the nuances of our dataset and the finding that normalization, when applied to hematoxylin and eosin-stained tissue images, does not always result in improved model performance.[19] In cases where stain normalization can be feasibly applied, common techniques include Reinhard, Ruifrok, Macenko, and Vahadane stain normalization. These techniques rely on a consistent ratio between slide background and tissue within each image, or for the image to be nearly solely comprised of tissue, both of which were not practical for the purposes of this study. Adaptive color normalization is *not* applied to the patches due to noise from artifacts and zero-padding, which skew pixel value histograms during target-image matching.

Table 2. Slide and Section Details

	Count (%)
Patients	25
Slides	229
Sections	1302
Patches	24,372
Normal sections	766 (58.8%)
Abnormal sections	536 (41.2%)
Containing active inflammation (% of abnormal, % of total)	468 (87.3%, 35.9%)
Containing chronic changes (% of abnormal, % of total)	426 (79.5%, 32.7%)

Cross-validation

Five-fold cross-validation was implemented. In every fold, scans from 20 patients were used for training and validation, while scans from the remaining 5 patients were used for testing, corresponding to approximately 1042 scans used for training and validation, and 260 scans for testing in each fold. Patients were randomly assigned to training, validation, and testing cohorts, and all scans for a patient were assigned to a single cohort with no overlap. Within each training/validation cohort, scans from 3 patients were used for validation. The maximum number of patches generated from a single section scan was 90, while the minimum number was 5. No tissue sections were discarded for having too few usable patches. This was done to accommodate the real-world scenario of variations in tissue acquisition. The numbers of tissue section scans for each fold by training/validation and testing cohorts are provided in Supplementary Table 2.

Multiple Instance Learning

Variations of multiple instance learning (MIL) are used for histopathology imaging classification tasks due to the large size of the data and infeasibility associated with inputting an entire section image into the model.[20–23] MIL is a form of weakly supervised learning; predictions are made on unlabeled instances that together comprise an overall labeled data point.

For this study, the dataset is composed of tissue section scans where each scan contains a set of smaller image patches. Generally, a scan containing at least one abnormal patch should be labeled as abnormal. MIL models predict scan labels without knowledge of individual patch labels. For example, a scan labeled as containing active inflammation contains at least one patch with active inflammation. In this study, there were no ground truth labels for individual patches, since labeling was performed on the tissue section scans. The specific patches containing active inflammation are unknown for training, validation, and testing sets.

Computer Vision Models for Tissue Classification

Three customized two-dimensional (2D) CNNs were developed to perform patch classification. Transfer learning and/or common deep learning architectures were not utilized as is typical for similar studies.[14,23,24] These custom models performed well and were suitable for each task. The first model distinguished between normal and abnormal tissue sections. The second and third models indicated whether a tissue section contained active inflammation and chronic changes, respectively. Figure 2 is a schematic of the pipeline from preprocessing to overall tissue section classification based on patch aggregation.

The models consisted of 4 convolutional layer sets. Each set started with a 2D convolutional layer (Conv2D) with kernel size of (3,3) and exponential linear unit activation, followed by batch normalization, and 2D maximum pooling (MaxPool2D) with stride of (2,2) and dropout. The number of filters for each consecutive set was 16, 32, 64, and 128. Dropout for each set was 0.1, 0.15, 0.2, and 0.25. An L1 regularization penalty of 0.0001 was applied to each Conv2D layer kernel to prevent overfitting.[25] A 2D global max pooling layer, dense layer with 256 neurons, and dropout of 0.3 followed the final convolutional layer set. The last layer was a dense layer with a single output and sigmoid activation function. Adam[26] optimizer with a learning rate of 0.0001 and binary cross entropy loss were utilized. The $128 \times 128 \times 3$ image patches were input into the model in batches of size 4. The best model weights in terms of validation accuracy were saved. Early stopping was implemented at a patience of 50 epochs, with 300 epochs set as the maximum.

All code was written in Python 3.10[27,28] with TensorFlow 2.11.1[29] implementation of Keras 2.11.0[30], and OpenSlide[31] version 1.2.0.

Considerations for Bias

Scan-level predictions were determined by aggregating patch-level predictions. If more than 50% of patches from a

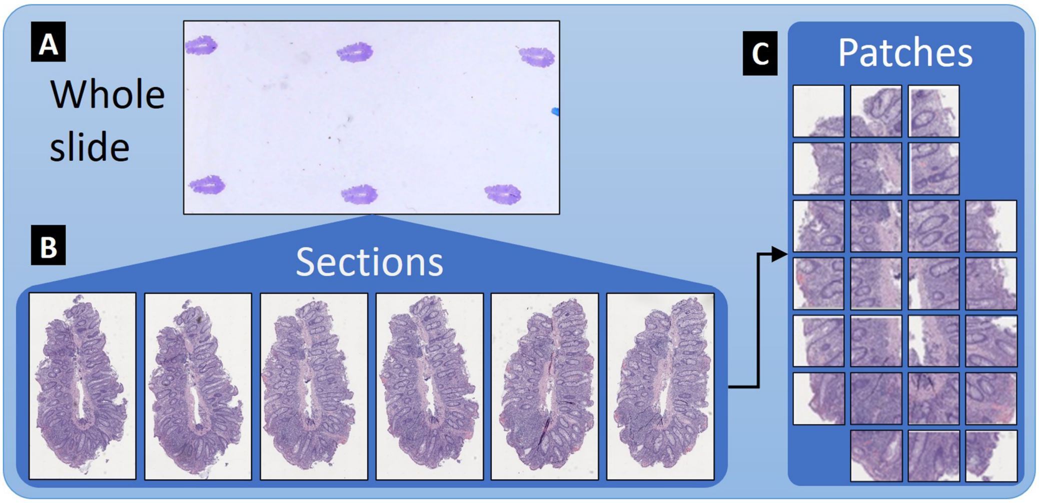

Figure 1. (A) Representative whole slide (≈9 slides per patient). (B) Six tissue section images obtained from the whole slide represented in (A) (≈6 tissue sections per slide). (C) Usable patches from a single tissue section (≈19 usable patches per tissue section).

scan were predicted to be abnormal (positive), with $P > .5$ being the requirement for abnormal classification of a patch, then the overall scan was classified as abnormal. There were no ground truth labels for individual patches since labeling was performed on the tissue section scans. Although it seems reasonable to designate a scan as abnormal if it contains just 1 or 2 abnormal patches, a threshold of 50% was utilized. This was due to the bias associated with labeling all patches from an abnormal scan as abnormal during training, though most patches, even from an abnormal scan, did not contain any abnormal tissue.

Limitations of this study include introduction of noise into the dataset due to an imperfect preprocessing pipeline that did not remove all image patches with pervasive artifacts and inadequate amounts of tissue. We estimate that approximately

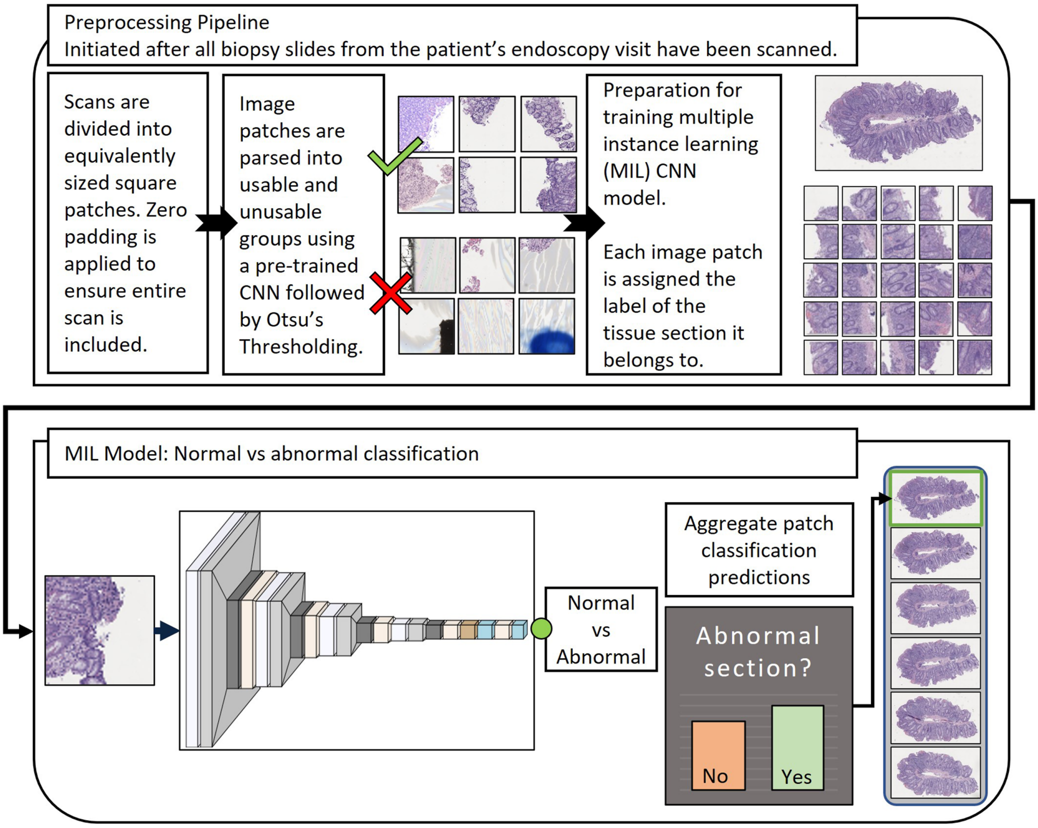

Figure 2. Workflow for tissue section scan preprocessing and patch prediction CNN with aggregation for overall normal vs abnormal classification of the tissue section.

Table 3. Model Performance

	Abnormal	Active inflammation	Chronic changes
Accuracy (SD)	0.839 (0.068)	0.851 (0.030)	0.858 (0.056)
Balanced accuracy (SD)	0.824 (0.068)	0.826 (0.032)	0.816 (0.073)
Precision (SD)	0.855 (0.115)	0.828 (0.089)	0.843 (0.085)
Sensitivity (SD)	0.735 (0.087)	0.739 (0.077)	0.695 (0.148)
Specificity (SD)	0.913 (0.073)	0.914 (0.049)	0.937 (0.033)
MCC (SD)	0.666 (0.149)	0.671 (0.074)	0.668 (0.135)
F1-score (SD)	0.790 (0.080)	0.781 (0.043)	0.762 (0.095)
ROC-AUC (SD)	0.915 (0.063)	0.917 (0.028)	0.929 (0.040)

SD, standard deviation.

5% of the patches in the dataset used for training, validation, and testing contained less than 20% tissue, including patches with no tissue. Furthermore, the data utilized for this study are limited to a single institution; therefore, there is justification for determining model robustness when applied to similar datasets from additional institutions.

Results

The abnormal vs normal classification CNN MIL model achieved an accuracy of 0.84, an area under the receiver operating characteristic curve (AUC-ROC) of 0.91, and an F1-score of 0.79 and 0.67. The accuracy for predicting active inflammation was 0.85, AUC-ROC was 0.92, and F1-score was 0.78. The accuracy for predicting chronic changes was 0.86, with an AUC-ROC of 0.93 and an F1-score of 0.76. The Matthews correlation coefficient (MCC) for all 3 models was 0.67. Model performance is most comprehensible when several metrics are provided since metric values on their own can be misleading. The MCC has been included as an important binary classification metric since it encompasses sensitivity, specificity, precision, and negative predictive value into a single value.[32] The MCC ranges from −1 to 1, where 1 corresponds to perfect classification, 0 corresponds to predictions made by chance, and −1 corresponds to perfect negative classification. MCC values for all 3 models utilized in this study indicated strong classification prediction given class imbalance. The full results for each task are provided in Table 3. Confusion matrices and AUC-ROC plots for the 3 tissue classifiers are provided in Figure 3. Granuloma classification was not considered due to extreme class imbalance within the dataset.

Training took 22 seconds per epoch (5 ms per step) on ml.g5.8xlarge AWS SageMaker training instance (instance resource availability: single GPU, 32 virtual CPUs, 128 GiB memory, 24 GiB GPU memory, 1 × 900 NVMe SSD instance storage). Patch classification using the trained CNN MIL model was instantaneous for each test set; approximately 7.59 ms per patch.

Gradient-weighted Class Activation Mapping[33] is often utilized as a means of providing model interpretability. Figure 4 presents correctly classified abnormal and normal patches with associated Gradient-weighted Class Activation Mapping to indicate regions of importance per the AI model. In abnormal patches, the model tended to focus on increased cellularity within the lamina propria including neutrophilic infiltration and lymphoplasmacytosis. These features are indeed indicative of abnormal tissue. Normal patches contained crypts having normal morphology and no increased lamina propria cellularity. Thus, these patches did not meet the model's threshold for abnormal classification and were correctly classified as normal.

Discussion

The 3 models performed well, suggesting that AI applied to images of histopathology tissue can assist in the diagnosis of IBD. The findings resulting from this study are significant primarily because they indicate that there is a strong AI-interpretable signal present in endoscopic WSI.

This research is unique for several reasons. First, the proposed models do not require patches from sections to be labeled for training or require that only preselected regions of interest (ROIs) be utilized. The authors of Chernavskaia et al[12] mention that their study required annotation of ROIs by a trained pathologist to facilitate image processing and prediction since implementation on the entire sample did not produce accurate prediction of histological indices. Similar efforts were performed in Gupta et al to annotate normal and abnormal regions.[34] In Bejnordi et al,[15] which implements context-aware stacked CNNs for classification of breast carcinomas, image patches were generated by random selection of samples from points inside contours of pathologist-segmented regions for each class. Our approach is amenable to implementation in clinical workflow because scans can be inputted into the preprocessing pipeline as is, automatically patched and parsed, and then inputted into the classification model without intervention from the user.

Second, predictions apply to more than abnormality. Classification further pertains to the presence of certain phenotypes in the tissue despite an individual patient's diagnosis. Additionally, overall predictions pertaining to the presence of abnormal tissue, active inflammation, and chronic changes are made at the section level (Part B of Figure 1), thus pinpointing the specific slice of tissue on the slide that the pathologist should examine, which clinicians

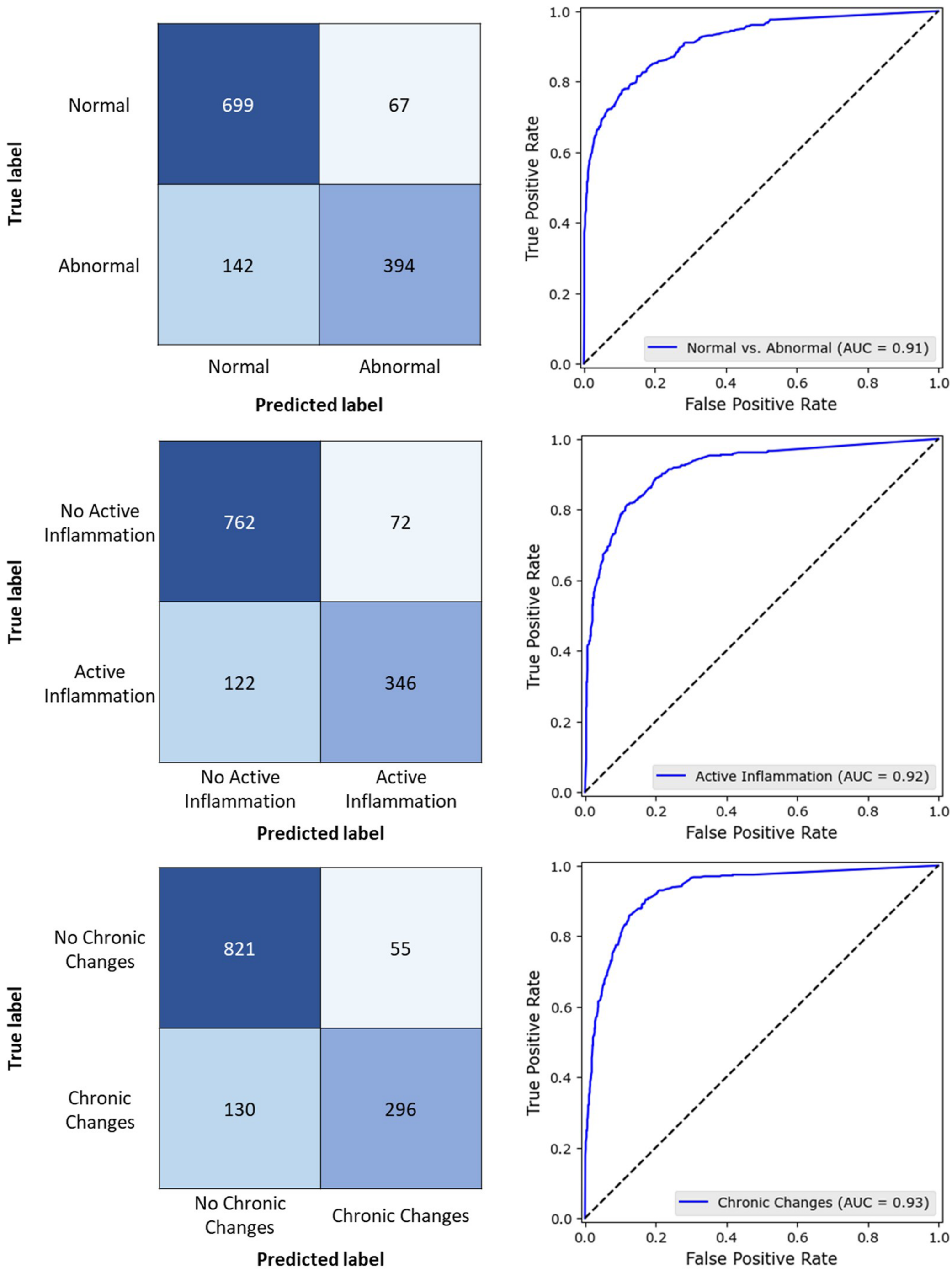

Figure 3. Confusion matrices and AUC-ROC plots for the 3 tissue classifiers.

can then use to determine diagnosis. Classification in similar studies is often at the slide level.[14,23,24,35]

Third, our sample population includes both CD and UC on a pediatric population and there is no assumption that the patient is one or the other. ML- and AI-based studies that incorporate histologic features tend to pertain specifically to UC and/or adult cohorts.[12–15] Clinically, it is difficult to distinguish between the 2 diagnoses; therefore, both have been included in this study since solely focusing on patients with one type of IBD and not the other could introduce bias.

Image data preprocessing for our study entails a pipeline to remove automatically generated image patches with insufficient amounts of tissue. Generating patches, or tiles, is a standard preprocessing step when working with large slide images. Preselected ROIs were not used to train our models. Assessing model results when model input requires user-generated ROIs or patches produces an incomplete understanding of model performance on realistic data for which such manual initial steps cannot be feasibly performed.

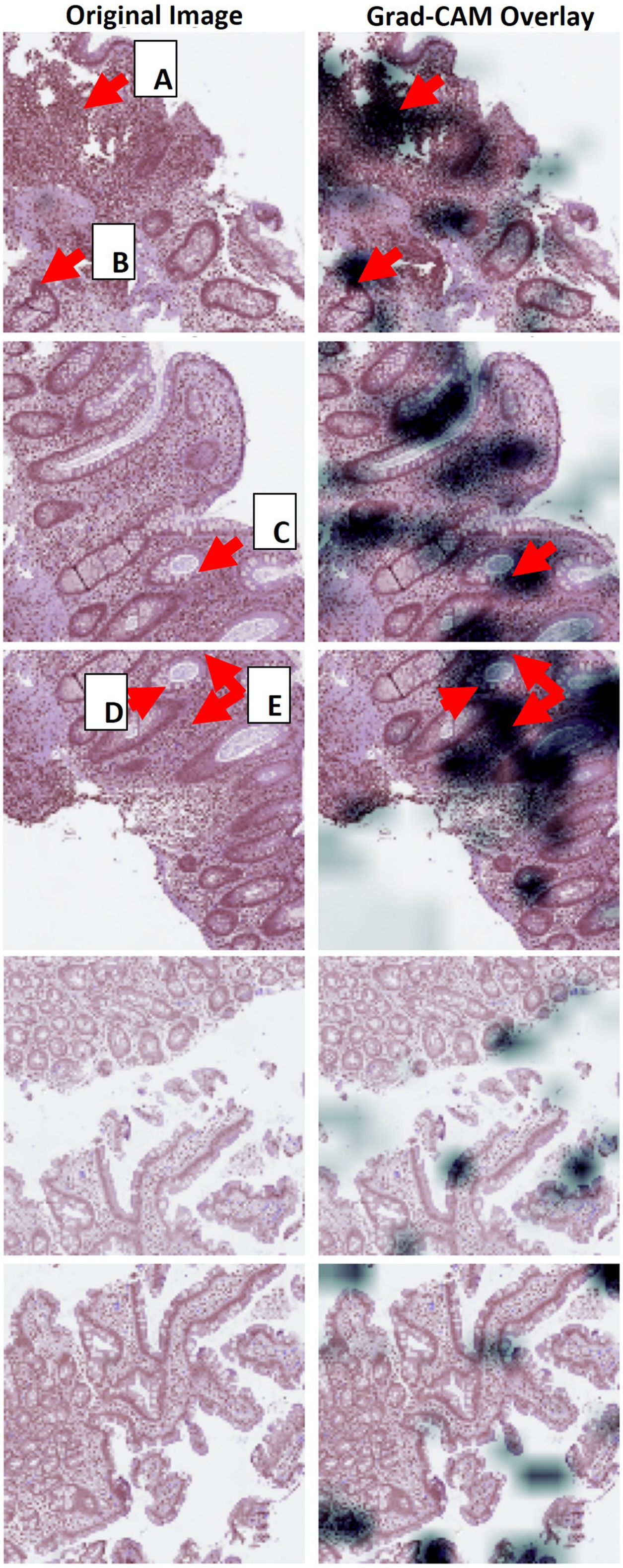

Figure 4. Abnormal patches (top 3 image sets): (A) and (E) Dense inflammatory infiltrates. (B–D) Neutrophilic infiltration within the wall of the crypt indicating cryptitis. Normal patches (bottom 2 image sets): Sparse overlay. The crypts have normal morphology and there is no increased lamina propria cellularity.

Future improvements to this work include further developing the automatic preprocessing pipeline for removing image patches with insufficient amounts of tissue. Additionally, a larger patient cohort would lead to more robust classification models and preprocessing CNN. Given the importance of the presence of granulomas in the assessment of CD severity, future work stemming from this study will utilize more patients, thus increasing the number of CD patient samples and tissue sections containing granuloma.

The opportunity to incorporate this histologic dataset into a model that predicts patient outcomes and ultimately expands to include many of the common variables seen in clinical care lies in the future. The possibility that sufficient data can be aggregated to help identify the nuances of pediatric IBD at the patient-specific level is a clinical care goal.

Supplementary Materials

Material associated with this article can be found in the online version at https://doi.org/10.1016/j.gastha.2026.100899.

References

1. Ananthakrishnan AN. Epidemiology and risk factors for IBD. Nat Rev Gastroenterol Hepatol 2015;12:205–217.
2. Loddo I, Romano C. Inflammatory bowel disease: genetics, epigenetics, and pathogenesis. Front Immunol 2015;6.
3. Van Limbergen J, Radford-Smith G, Satsangi J. Advances in IBD genetics. Nat Rev Gastroenterol Hepatol 2014;11:372–385.
4. Bonen DK, Cho JH. The genetics of inflammatory bowel disease. Gastroenterology 2003;124:521–536.
5. Lakatos PL. Environmental factors affecting inflammatory bowel disease: have we made progress? Dig Dis 2009;27:215–225.
6. Carbonnel F, Jantchou P, Monnet E, et al. Environmental risk factors in Crohn's disease and ulcerative colitis: an update. Gastroenterol Clin Biol 2009;33:S145–S157.
7. Dhaliwal J, Walters T, Mack D, et al. Phenotypic variation in paediatric inflammatory bowel disease by age: a multicentre prospective inception cohort study of the Canadian Children IBD Network. J Crohns Colitis 2020;14:445–454.
8. Ruel J, Ruane D, Mehandru S, et al. IBD across the age spectrum—is it the same disease? Nat Rev Gastroenterol Hepatol 2014;11:88–98.
9. Tontini GE, Vecchi M, Pastorelli L, et al. Differential diagnosis in inflammatory bowel disease colitis: state of the art and future perspectives. World J Gastroenterol 2015;21:21.
10. Feakins R, Borralho Nunes P, Driessen A, et al. Definitions of histological abnormalities in inflammatory bowel disease: an ECCO position paper. J Crohns Colitis 2024;18:175–191.
11. Kővári B, Báthori Á, Friedman MS, et al. Histologic diagnosis of inflammatory bowel diseases. Adv Anat Pathol 2022;29:48–61.
12. Chernavskaia O, Heuke S, Vieth M, et al. Beyond endoscopic assessment in inflammatory bowel disease:

real-time histology of disease activity by non-linear multimodal imaging. Sci Rep 2016;6:29239.
13. Najdawi F, Sucipto K, Mistry P, et al. Artificial intelligence enables quantitative assessment of ulcerative colitis histology. Mod Pathol 2023;36:100124.
14. del Amor R, Meseguer P, Parigi T, et al. Constrained multiple instance learning for ulcerative colitis prediction using histological images. Comput Methods Programs Biomed 2022;224:107012.
15. Bejnordi BE, Zuidhof G, Balkenhol M, et al. Context-aware stacked convolutional neural networks for classification of breast carcinomas in whole-slide histopathology images. J Med Imaging 2017;4:1.
16. Martin-King C, Nael A, Ehwerhemuepha L, et al. Histopathology imaging and clinical data including remission status in pediatric inflammatory bowel disease. Sci Data 2024;11:761.
17. Cohen J. Weighted kappa: nominal scale agreement with provision for scaled disagreement or partial credit. Psychol Bull 1968;70:213–220.
18. Landis JR, Koch GG. The measurement of observer agreement for categorical data. Biometrics 1977; 33:159–174.
19. Hameed Z, Garcia-Zapirain B, Aguirre JJ, et al. Multiclass classification of breast cancer histopathology images using multilevel features of deep convolutional neural network. Sci Rep 2022;12:15600.
20. Ilse M, Tomczak JM, Welling M. Attention-based deep multiple instance learning. arXiv; 2018. http://doi.org/10.48550/arXiv.1802.04712.
21. Wang J, Mao Y, Guan N, et al. Advances in multiple instance learning for whole slide image analysis: techniques, challenges, and future directions. arXiv; 2024. http://doi.org/10.48550/arXiv.2408.09476.
22. Bazargani R, Fazli L, Gleave M, et al. Multi-scale relational graph convolutional network for multiple instance learning in histopathology images. Med Image Anal 2024;96:103197.
23. Sharma AH, Lawlor BW, Wang JY, et al. Deep learning for predicting pediatric Crohn's disease using histopathological imaging. In: 2022 Systems and Information Engineering Design Symposium (SIEDS); 2022:122–127. Institute of Electrical and Electronics Engineers Inc.
24. Sali R, Adewole S, Ehsan L, et al. Hierarchical deep convolutional neural networks for multi-category diagnosis of gastrointestinal disorders on histopathological images. In: 2020 IEEE International Conference on Healthcare Informatics (ICHI); 2020:1–6. Institute of Electrical and Electronics Engineers Inc.
25. Thanapol P, Lavangnananda K, Bouvry P, et al. Reducing overfitting and improving generalization in training convolutional neural network (CNN) under limited sample sizes in image recognition. In: Proceedings of the 5th International Conference on Information Technology (InCIT); October 21-22, Bangsaen, Thailand. IEEE; 2020:300–305.
26. Kingma DP, Ba J. Adam: a method for stochastic optimization. arXiv; 2017. http://doi.org/10.48550/arXiv.1412.6980.
27. Python Software Foundation. Python language reference, version 3.9. http://www.python.org. Accessed July 14, 2025.
28. van Rossum G. Python tutorial, Technical Report CS-R9526, Centrum voor Wiskunde en Informatica (CWI), Amsterdam. CWI; 1995:620.
29. Abadi M, Barham P, Chen J, et al. TensorFlow: a system for large-scale machine learning TensorFlow: a system for large-scale machine learning. 2015. https://tensorflow.org. Accessed July 14, 2025.
30. Chollet F. Keras. 2015. https://keras.io. Accessed July 14, 2025.
31. Goode A, Gilbert B, Harkes J, et al. OpenSlide: a vendor-neutral software foundation for digital pathology. J Pathol Inform 2013;4:27.
32. Chicco D, Jurman G. The Matthews correlation coefficient (MCC) should replace the ROC AUC as the standard metric for assessing binary classification. BioData Min 2023;16:4.
33. Selvaraju RR, Cogswell M, Das A, et al. Grad-CAM: visual explanations from deep networks via gradient-based localization. Int J Comput Vis 2019;128:336–359.
34. Gupta P, Huang Y, Sahoo P, et al. Colon tissues classification and localization in whole slide images using deep learning. Diagnostics 2021;11:1398.
35. Iacucci M, Parigi T, Del Amor R, et al. Artificial intelligence enabled histological prediction of remission or activity and clinical outcomes in ulcerative colitis. Gastroenterology 2023;164:1180–1188.e2.
36. Martin-King C, Nael A, Ehwerhemuepha L, et al. Histopathology imaging and clinical data including remission status in pediatric inflammatory bowel disease. Sci Data 2024;11:761.

Received November 25, 2024. Accepted February 11, 2026.

Correspondence:
Address correspondence to: Chloe Martin-King, PhD, CHOC Children's, Research Institute, 1201 W. La Veta Avenue, Orange, California 92868. e-mail: chloe.martin.king@choc.org.

Acknowledgments:
Alex Grant extracted structured data from the 12- and 52-week encounters for each patient. Shih-hsin Kan trained the scanning team on how to use the MikroScan digital pathology scanner and software. Willy Wong is the software engineer for CIL and wrote the PHP script for downloading the 18-patient dataset subset.

Authors' Contributions:
Chloe Martin-King: Data curation, formal analysis, investigation, methodology, software, validation, visualization, writing - original draft, writing - review and editing. Ali Nael: Investigation, writing - review and editing. Louis Ehwerhemuepha: Conceptualization, writing - review and editing. Blake Calvo: Investigation, writing - review and editing. Quinn Gates: Methodology. Jamie Janchoi: Data curation, investigation, project administration. Elisa Ornelas: Data curation, investigation, project administration. Melissa Perez: Project administration. Andrea Venderby: Investigation. John Miklavcic: Supervision, writing - review and editing. Peter Chang: Methodology. Aaron Sassoon: Investigation. Brian Rubio: Investigation. Ghislaine Barragan: Investigation. Kenneth Grant: Conceptualization, funding acquisition, supervision, writing - original draft, writing - review and editing.

Conflicts of Interest:
The authors disclose no conflicts.

Funding:
The research in this article was supported internally by CHOC's CSO Small Grant Program. Furthermore, John Miklavcic, Blake Calvo, and Andrea Venderby received funding throughout the duration of the project by USDA NIFA under award number 2021-38420-34941.

Ethical Statement:
This retrospective study is considered minimal risk and was approved by CHOC's in-house institutional review board (IRB), IRB number 2111186. A waiver of informed consent was granted.

Data Transparency Statement:
Imaging and structured data for 18 of the 25 patients utilized in the experiments detailed in this article are publicly available on the Cell Image Library[36] (CIL) website. Full descriptions can be found in the associated data descriptor manuscript by Martin-King et al.[16]

Reporting Guidelines:
2024 updated CLAIM (Checklist for Artificial Intelligence in Medical Imaging).

Gastro Hep Advances, Volume 5

Supplemental information

Pediatric Inflammatory Bowel Disease Tissue Classification From Pathology Slide Images: Detecting Phenotypes Using Computer Vision

Chloe Martin-King, Ali Nael, Louis Ehwerhemuepha, Blake Calvo, Quinn Gates, Jamie Janchoi, Elisa Ornelas, Melissa Perez, Andrea Venderby, John Miklavcic, Peter Chang, Aaron Sassoon, Brian Rubio, Ghislaine Barragan, and Kenneth Grant

Supplemental table 1 Interobserver rater results and interobserver reliability for sections in additionally labelled subset

		Rater 2	
**Abnormal vs normal**		Abnormal	Normal
**Rater 1**	Abnormal	158	1
	Normal	4	11
**Active inflammation**		Present	Absent
**Rater 1**	Present	142	1
	Absent	14	17
**Chronic changes**		Present	Absent
**Rater 1**	Present	105	12
	Absent	15	42

	**κ (95% confidence interval)**
**Abnormal vs. normal**	0.799 (0.626, 0.973)
**Active inflammation**	0.648 (0.477, 0.818)
**Chronic changes**	0.643 (0.519, 0.767)

κ: Cohen's Kappa Statistic

Supplemental Table 2 Number of tissue section scans in each cohort

	Fold 1		Fold 2		Fold 3		Fold 4		Fold 5	
	**Train/Val**	**Test**	**Train/Val**	**Test**	**Train/Val**	**Test**	**Train/Val**	**Test**	**Train/Val**	**Test**
**Total scans**	1018 (0.78)	284 (0.22)	1062 (0.82)	240 (0.18)	1093 (0.84)	209 (0.16)	1002 (0.77)	300 (0.23)	1033 (0.79)	269 (0.21)
**Normal**	614 (0.60)	152 (0.54)	602 (0.57)	164 (0.68)	651 (0.60)	115 (0.55)	591 (0.59)	175 (0.58)	606 (0.59)	160 (0.59)
**Abnormal**	404 (0.40)	132 (0.46)	460 (0.43)	76 (0.32)	442 (0.40)	94 (0.45)	411 (0.41)	125 (0.42)	427 (0.41)	109 (0.41)
**No inflam**	657 (0.65)	177 (0.62)	669 (0.63)	165 (0.69)	713 (0.65)	121 (0.58)	641 (0.64)	193 (0.64)	656 (0.64)	178 (0.66)
**Inflam**	361 (0.35)	107 (0.38)	393 (0.37)	75 (0.31)	380 (0.35)	88 (0.42)	361 (0.36)	107 (0.36)	377 (0.36)	91 (0.34)
**No changes**	685 (0.67)	191 (0.67)	705 (0.66)	171 (0.71)	754 (0.69)	122 (0.58)	675 (0.67)	201 (0.67)	685 (0.66)	191 (0.71)
**Changes**	333 (0.33)	93 (0.33)	357 (0.34)	69 (0.29)	339 (0.31)	87 (0.42)	327 (0.33)	99 (0.33)	348 (0.34)	78 (0.29)
